# Vitamin C and Neutrophil Function: Findings from Randomized Controlled Trials

**DOI:** 10.3390/nu11092102

**Published:** 2019-09-04

**Authors:** Mikee Liugan, Anitra C. Carr

**Affiliations:** 1 Centre for Postgraduate Nursing Studies, University of Otago, Christchurch 8011, New Zealand; 2 Nutrition in Medicine Research Group, Department of Pathology & Biomedical Science, University of Otago, Christchurch 8011, New Zealand

**Keywords:** vitamin C, ascorbic acid, neutrophils, polymorphonuclear leukocytes, migration, chemotaxis, apoptosis, phagocytosis, oxidative burst, systematic review

## Abstract

Vitamin C is known to support immune function and is accumulated by neutrophils to millimolar intracellular concentrations suggesting an important role for the vitamin in these cells. In this review, the effects of vitamin C, as a mono- or multi-supplement therapy, on neutrophil function were assessed by conducting a systematic review of randomized controlled trials (RCTs). Specifically, trials which assessed neutrophil migration (chemotaxis), phagocytosis, oxidative burst, enzyme activity, or cell death (apoptosis) as primary or secondary outcomes were assessed. A systematic literature search was conducted using the Cochrane Central Register of Controlled Trials, EMBASE, Embase Classic, Joanna Briggs Institute EBP, Ovid MEDLINE^®^, Ovid MEDLINE^®^ In-Process & Other Non-Indexed Citations, Ovid Nursing Database, CINAHL and PubMed database, which identified 16 eligible RCTs. Quality appraisal of the included studies was carried out using the Cochrane Risk of Bias tool. Three of the studies assessed neutrophil chemotaxis in hospitalised patients or outpatients, two of which showed improved neutrophil function following intravenous vitamin C administration. Ten RCTs assessed neutrophil phagocytosis and/or oxidative burst activity; five were exercise studies, one in smokers, one in myocardial infarction patients and three in healthy volunteers. Two of the multi-supplement studies showed a difference between the intervention and control groups: increased oxidative burst activity in athletes post-exercise and decreased oxidant generation in myocardial infarction patients. Two studies assessed neutrophil enzyme activity; one showed deceased antioxidant enzyme activity in divers and the other showed increased antioxidant enzyme activity in athletes. One final study showed decreased neutrophil apoptosis in septic surgical patients following intravenous vitamin C administration. Overall, 44% of the RCTs assessed in this review showed effects of vitamin C supplementation on neutrophil functions. However, the studies were very heterogeneous, comprising different participant cohorts and different dosing regimens. There were also a number of limitations inherent in the design of many of these RCTs. Future RCTs should incorporate prescreening of potential participants for low vitamin C status or utilize cohorts known to have low vitamin status, such as hospitalized patients, and should also comprise appropriate vitamin C dosing for the cohort under investigation.

## 1. Introduction

Neutrophils are a vital component of the innate immune system, providing a first line of defense against invading pathogens [1]. Following microbial invasion, neutrophils migrate to the site of infection in response to pathogen- and host-derived pro-inflammatory mediators, known as chemotaxis [1]. The neutrophils then proceed to phagocytose, kill and digest the invading pathogens via both oxidative and enzymatic mechanisms [2]. Spent neutrophils subsequently undergo a process of programmed cell death which results in recognition and clearance of the cells by macrophages [3]. Effective clearance of neutrophils from inflammatory loci is vital for resolution of the pro-inflammatory response as release of necrotic cell contents results in tissue damage [4]. Chromatin released from neutrophils, known as neutrophil extracellular traps, comprises both oxidative and proteolytic enzymes, and has been implicated in host tissue damage and various pathologies [5].

Defective neutrophil function is observed in a number of conditions, such as chronic granulomatous disease and Chédiak-Higashi syndrome, which result in recurrent infections [6,7]. Patients with recurrent infections and sepsis can also present with dysfunctional neutrophils, sometimes referred to as immune paralysis due to the inability of the cells to migrate appropriately [8]. It is noteworthy that patients with severe infections and sepsis present with depleted vitamin C status [9,10]. Vitamin C is known to have pleiotropic roles in the immune system, through its antioxidant and enzyme cofactor activities, including potentially supporting neutrophil function [11]. Preclinical studies indicate that neutrophils isolated from scorbutic guinea pigs exhibit attenuated chemotaxis, phagocytosis, oxidant production and microbial killing compared with control animals, and supplementation with vitamin C reversed the dysfunctional activities [12,13,14]. Vitamin C-deficient gulonolactone oxidase (Gulo) knockout mice exhibit dysfunctional neutrophil cell death and diminished uptake by macrophages [15], and vitamin C supplementation can decrease neutrophil extracellular traps formation in septic Gulo knockout mice [16].

Although mean plasma vitamin C concentrations are typically around 50 µmol/L, neutrophils accumulate millimolar intracellular vitamin C concentrations against a concentration gradient which is thought to indicate an important role for the vitamin in these cells [17]. Thus, the depleted vitamin C status of neutrophils observed during infectious episodes could potentially compromise their function [18]. Numerous non-controlled studies have investigated the effects of vitamin C supplementation on the neutrophil functions of chemotaxis, phagocytosis, oxidant generation and microbial killing and predominantly showed positive effects (reviewed in [11]). A number of these studies included patients with known neutrophil dysfunction e.g., those with chronic granulomatous disease or Chédiak-Higashi syndrome, or individuals with allergic or infectious conditions. Exercise, both single bouts and prolonged training over several weeks, can produce changes in the distribution and function of various cellular and humoral components of the immune system [19]. Studies have reported high susceptibility of athletes to infections, especially upper respiratory tract infections, following heavy and intensive training as well as after marathon and ultramarathon running [20,21]. Thus, the effect of vitamin C supplementation on neutrophil function in athletes is also of interest.

The purpose of this review was to identify RCTs which investigated the effects of vitamin C supplementation on the functions of neutrophils. These included RCTs in athletes, healthy volunteers and patient groups, but excluding participants with existing neutrophilic dysfunction disorders. Studies comprising vitamin C administered as monotherapy or in combination with other micronutrients, such as vitamin E, were included. No restriction was placed on the route of administration (oral or intravenous) or the source of vitamin C (supplemental or food-derived) [22].

## 2. Methodology

The research question for this review was formulated using the PICO tool which comprises Population, Intervention, Comparison and Outcomes, an approach endorsed by the Cochrane Collaboration [23]. The PICO question was: What are the effects of vitamin C supplementation on neutrophil function, particularly on neutrophil chemotaxis/motility/migration, phagocytosis, oxidative burst, enzyme activity, apoptosis/clearance or necrosis/necrotic cell death in humans?

Preliminary literature searches were conducted using the databases PROSPERO, DARE, NICE, and the Cochrane Database of Systematic Reviews to determine any existing literature available on the topic area, to identify any existing or ongoing reviews of relevance to the topic area to ensure that the topic to be reviewed was novel and that there were no published reviews with the same research question in the current literature. A thorough literature search was then conducted using the Cochrane Central Register of Controlled Trials (May 2018), EMBASE (1980 to June 2018), Embase Classic (1947 to 1979), Joanna Briggs Institute EBP (June 2018), Ovid MEDLINE^®^ In-Process & Other Non-Indexed Citations (June 2018), Ovid MEDLINE^®^ (1946 to Present with Daily Update), Ovid Nursing Database (1946 to May 2018), CINAHL and PubMed databases using the following keywords: (1) vitamin C OR ascorbic acid OR ascorbate OR antiscorbutic factor OR l-ascorbate OR l-ascorbic acid; AND (2) neutrophils OR polymorphonuclear leukocytes OR immune system OR immunity OR immune function OR inflammation; AND (3) migration OR mobility OR chemotaxis OR apoptosis OR phagocytosis OR clearance OR necrosis OR necrotic cell death OR oxidative burst OR neutrophil enzyme activity. No restrictions were placed for the publication date, study location, age of participants, nature of participants, route of administration of vitamin C, and the source of vitamin C (supplemental or food-derived). However, only published RCTs and publications in English were included in this review. 

The abstracts and titles of the papers obtained from the literature search were screened to ensure that all the PICO components were covered and also that the inclusion/exclusion criteria were met (Table 1). Studies that did not meet the inclusion/exclusion criteria were rejected. Full-texts of the relevant papers were obtained using the University of Otago library and ResearchGate websites. Each of the full-text articles gathered for analysis were critically appraised for quality and risk of bias using the Cochrane Risk of Bias tool for RCTs [23]. For every criterion, each study was rated either low, unclear or high risk of bias and therefore appraised as good, fair or poor-quality research. The primary outcome measure for this review was the relationship between vitamin C and neutrophil function in humans.

## 3. Results

### 3.1. Literature Search Outcome

**Figure 1:**
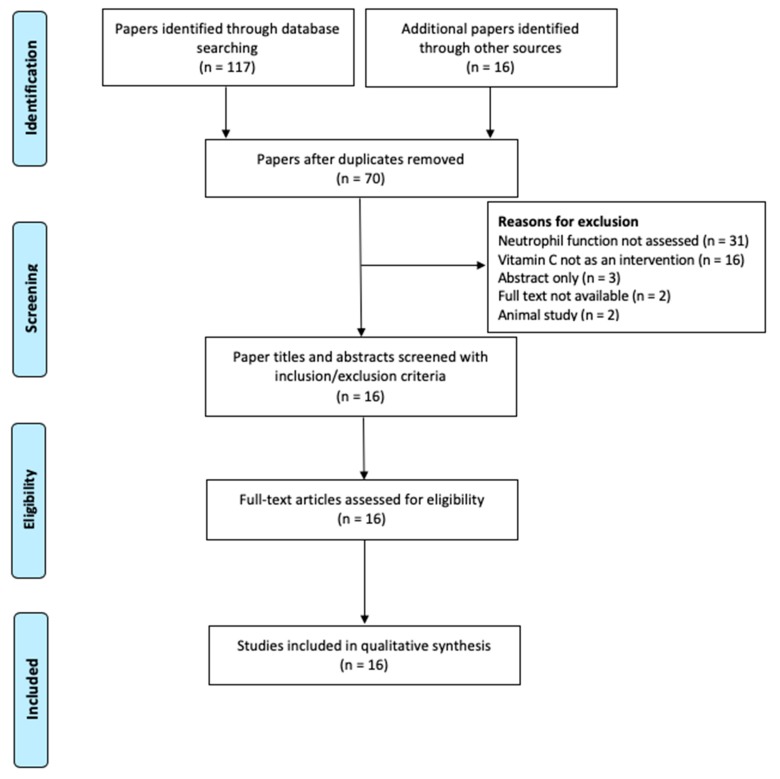
The study selection process presented using the PRISMA flow chart.

**Figure 2:**
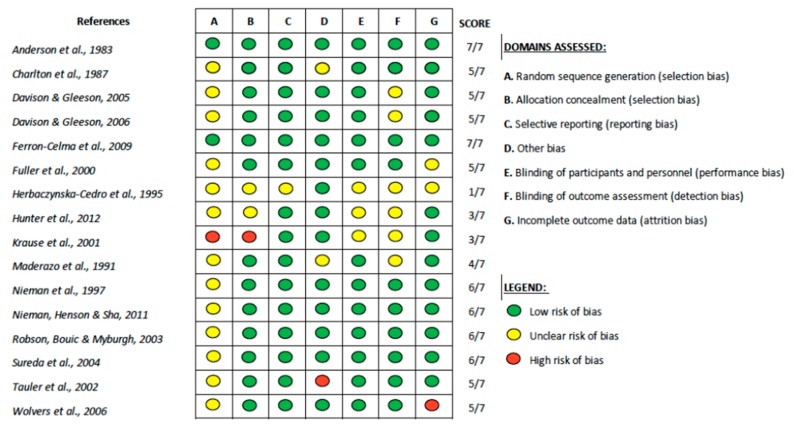
Quality appraisal results of the included studies using the Cochrane Risk of Bias tool.

The process of selection and screening of studies is illustrated in Figure 1 using the PRISMA flow chart [24]. Screening was based on the predetermined inclusion/exclusion criteria as well as the PICO components. After the screening process, sixteen papers were found to be eligible and were quality appraised for risks of bias using the Cochrane Risk of Bias tool (Figure 2) [23]. Four publications were rated low-quality following the critical appraisal, of which two papers failed to meet allocation concealment and blinding requirements scoring as high-risk for selection bias and performance bias while the other two papers had flawed statistical analysis owing to missing data thus scoring as high-risk for attrition bias and other bias. Due to the limited number of studies available, these four publications were included in the analysis and results, however, the limitations of these papers were highlighted. Thus, sixteen papers were included in the synthesis.

### 3.2. Study Characteristics

The 16 studies described were conducted in nine locations. These included Africa (*n* = 1), Austria (*n* = 1), New Zealand (*n* = 1), the Netherlands (*n* = 1), Poland (*n* = 1), Spain (*n* = 3), South Africa (*n* = 1), United States (*n* = 4), and the United Kingdom (*n* = 3). The 16 studies comprised nine that used vitamin C as monotherapy and seven that used combination supplementation. As illustrated in Table 2a,b (vitamin C only and combination studies, respectively), participants were recruited from a number of different settings including schools, clinics, communities, and surgical departments of hospitals. However, some studies did not specify the trial settings. The time frames of the studies ranged from one day to six-month long trials. The sample size of the studies ranged from six to 131 participants garnering a total number of 497 participants. There was a wide variation in the methodologies used between studies, e.g., the neutrophil assays were either carried out with whole blood or the cells were isolated prior to analysis in buffer or culture media (Table 2a,b), the intervention was administered at different doses, via different routes of administration (four studies used intravenous vitamin C, with trauma, surgical, and septic cohorts), and the treatment periods also varied dramatically from one day to six months (Table 3a,b). The included studies also covered a wide range of age groups as illustrated in Table 4. Furthermore, the control measures instigated for potential confounding factors were also variable, e.g., not all studies measured the plasma vitamin C concentrations at baseline, and diet control as well as smoking status were often not taken into consideration or reported (Table 4). Overall, three studies assessed neutrophil chemotaxis (with one also assessing phagocytosis), ten studies assessed neutrophil phagocytosis and/or oxidative burst activity, two studies assessed neutrophil enzyme activity, and one study assessed neutrophil apoptosis.

### 3.3. Outcomes

#### 3.3.1. Neutrophil Chemotaxis

Three of the included RCTs assessed neutrophil migration in response to chemoattractants; two were hospitalized cohorts (surgical premedication and trauma) and one from an outpatient clinic, and all three used intravenous vitamin C [25,26,27]. Anderson et al. [25] showed that administration of intravenous vitamin C (1000 mg once daily) to asthmatic children was associated with improved neutrophil chemotaxis compared with standard anti-asthma chemoprophylaxis (SAC) treatment alone. Charlton et al. [26] showed that premedication (papaveretum and hyoscine) used prior to surgery caused a decline in neutrophil chemotaxis in children undergoing elective surgical procedures. After the premedication was administered, neutrophil chemotaxis still fell significantly in the vitamin C group (single dose of 10 mg/kg) and was not significantly different to the placebo group. This suggests that vitamin C supplementation before premedication does not protect against depression of neutrophil chemotaxis in children as a result of the premedication given. 

Neutrophil chemotaxis following serious blunt trauma was shown to be significantly decreased soon after injury, reaching maximum depression by day two of injury [27]. Plasma vitamin C concentrations also decreased to half initial levels. Neutrophil chemotaxis increased for those treated for a week with both vitamins C and E (200 and 50 mg/day, respectively). For the patients who received either vitamin C or E alone, there was a slightly better response than the placebo group, but this did not reach statistical significance, likely due to the low participant numbers (e.g., only three analysed in the vitamin C group). Thus, the combination of vitamins C and E together gave a better response than either vitamin C or E alone with regard to neutrophil chemotaxis. 

#### 3.3.2. Phagocytosis and Oxidative Burst

Five studies investigated the effects of either vitamin C alone or antioxidant combinations on exercise-induced changes in neutrophil oxidative burst activity and/or phagocytosis [19,28,29,31,35]. Nieman et al. [19] showed that exercise caused a significant increase in granulocyte phagocytosis and a decrease in granulocyte oxidative burst post-exercise. The vitamin C group (1 g/day for eight days) did not differ significantly from the placebo group which suggests that vitamin C supplementation had no effect on the exercise-induced changes in granulocyte phagocytosis and oxidative burst activity. Krause et al. [31] reported decreased phagocytosis and bactericidal activity of neutrophils isolated after high-intensity exercise (biathlon), although no change in intracellular reactive oxygen species generation was observed. There were no differences between the vitamin C supplementation (2 g for one week) and the placebo groups in this study. 

Davison and Gleeson [28] investigated the influence of acute vitamin C supplementation (3.4 g single dose) and/or carbohydrate ingestion on neutrophil degranulation and oxidative burst activity after a prolonged exercise trial. They found that neutrophil degranulation was significantly decreased in the vitamin C and carbohydrate + vitamin C groups post-exercise but did not differ to the placebo group. In addition, neither vitamin C nor carbohydrate had an effect on the decrease in neutrophil oxidative burst activity post-exercise. Therefore, in this study, vitamin C supplementation had no effect on neutrophil degranulation or oxidative burst activity post-exercise. In a similar study conducted by the same investigators [29], it was reported that both oxidative burst activity and neutrophil degranulation was decreased post-exercise. Vitamin C supplementation (1 g/day for two weeks) was no different to the placebo group and thus did not influence neutrophil oxidative burst or degranulation. 

In contrast to the vitamin C-alone supplementation studies, Robson et al. [35] showed that neutrophil oxidative burst was significantly higher in trained runners following prolonged exercise in the antioxidant group (with vitamin C 960 mg/day for one week) compared to the placebo group post-exercise. Thus, antioxidant combinations may provide improved neutrophil function in athletes compared to vitamin C monotherapy.

One study investigated the effects of vitamin C supplementation on neutrophil oxidative burst of smokers [30]. Smoking increases susceptibility to bacterial and viral infections and compromises the anti-microbial functions of neutrophils [40,41]. Furthermore, smoking is associated with lower plasma vitamin C concentrations [42,43,44,45]. Fuller et al. [30] supplemented healthy smokers with vitamin C (1 g/day for eight weeks) and/or vitamin E but found no difference in neutrophil superoxide production in any of the treatment groups compared with placebo, despite an increase in plasma vitamin C status (from 39 to 62 µmol/L). 

Ischemia/reperfusion injury can also result in increased production of reactive oxygen species, inducing oxidative stress, and is common in disease states such as stroke and myocardial ischemia [46]. In a study of patients with acute myocardial infarction, Herbaczynska et al. [34] found that supplementation with vitamins C and E (600 mg/day) decreased oxygen free radical generation by neutrophils. Thus, the combination of vitamins C and E appeared to exhibit antioxidant functions in these patients.

Two studies investigated the effects of micronutrient supplementation on neutrophil phagocytosis and oxidative burst activities in healthy individuals [37,38]. Wolvers et al. [38] found that the consumption of micronutrient (with ~375 mg/day of vitamin C for ten weeks) did not affect the phagocytosing or oxidative burst activities of neutrophils in any of the treatment groups of healthy volunteers. Nieman et al. [37] showed that breakfast cereal fortified with varying levels of micronutrients (low, medium and high groups containing 0.8, 20, and 100 mg/day of vitamin C respectively) given over two months to healthy children also did not affect the pattern of change in granulocyte phagocytosis or oxidative burst. Finally, Hunter et al. [36] supplemented healthy older adults with a vitamin C rich food (gold kiwifruit containing ~360 mg vitamin C daily for four weeks), however, this had no effect on neutrophil phagocytosing activity. Thus, micronutrient supplementation or fortification, or vitamin C-rich foods, do not appear to influence neutrophil function in healthy individuals.

#### 3.3.3. Neutrophil Enzyme Activities

The enzymes catalase, superoxide dismutase and glutathione peroxidase scavenge reactive oxygen species and are therefore markers of neutrophil antioxidant defence [47]. Two studies investigated the effect of vitamin C, either alone or in an antioxidant cocktail, on neutrophil enzyme activities [32,39]. A study of a group of divers showed that oxidative stress induced by hypoxia can affect neutrophil function, specifically neutrophil antioxidant defences [32]. They showed that a group of divers supplemented with vitamin C (1 g/day for seven days) had significantly lower catalase and glutathione peroxidase enzyme activities compared to the placebo group at post-dive as well as during recovery. One interpretation was that vitamin C supplementation may have resulted in a higher rate of non-enzymatic scavenging of reactive oxygen species and hence a decrease in the activity of the antioxidant enzymes. In contrast, in a group of athletes, Tauler et al. [39] showed that three months of antioxidant supplementation (which included 500 mg/day vitamin C for the last fifteen days) significantly increased neutrophil enzyme activities, particularly of catalase, superoxide dismutase, glutathione peroxidase and glutathione reductase, relative to the placebo group. Once again, an antioxidant cocktail appeared to be more effective than monotherapy in athletes.

#### 3.3.4. Neutrophil Apoptosis

One study investigated the effects of vitamin C supplementation on apoptosis in neutrophils isolated from the peripheral blood of septic abdominal surgery patients [33]. Although these investigators did not assess the vitamin C status of their patients, it is well known that low vitamin C status is a common feature of patients with sepsis [9]. Ferron-Celma et al. [33] found that administration of intravenous vitamin C (450 mg/day for six days) resulted in significantly lower levels of the pro-apoptotic factors caspase-3 and poly-ADP-ribose polymerase (PARP) in the vitamin C group compared to the placebo group. In addition, significantly higher levels of the anti-apoptotic factor (Bcl-2) were observed in the vitamin C group compared to the placebo group. Therefore, this study indicated that vitamin C supplementation may exert anti-apoptotic effects on the peripheral blood neutrophils of septic patients.

## 4. Discussion

Overall, nine of the 16 RCTs included in this review reported no effect of supplementation with vitamin C alone, or in combination with other micronutrients or antioxidants, on various neutrophil functions [19,26,28,29,30,31,36,37,38]. The seven studies which did show effects of supplementation on the neutrophil functions assessed (i.e., chemotaxis, oxidative burst activity, antioxidant enzyme activity and apoptosis) were in hospitalized patients or outpatients [25,27,33,34] or athletes [32,35,39]. None of the other studies carried out with healthy volunteers showed any effects of additional supplementation.

Eight of 10 RCTs showed no effect of supplementation on neutrophil phagocytosis and/or oxidative burst activity [19,28,29,30,31,36,37,38]. The two studies that showed an effect on oxidative burst activity used combination supplements and they showed opposite effects [34,35]. Herbaczynska-Cedro et al. [34] showed decreased oxidant burst activity in acute myocardial infarction patients treated with a combination of vitamins C and E. It is well known that vitamin C can recycle vitamin E and vitamin C has been proposed to interact with vitamin E in vivo, thus they appear to work synergistically [48]. Although another study also tested a combination of vitamins C and E, they saw no effect on oxidative burst activity [30]. However, this study was carried out in smokers and it is known that smoking causes enhanced oxidative stress and smokers require significantly higher intakes of vitamin C to reach the same circulating concentrations as non-smokers [49]. In contrast, Robson et al. [35] showed an increase in oxidative burst activity in trained runners supplemented with an antioxidant combination following prolonged exercise. Since the baseline vitamin C status of the participants was already at saturating levels (i.e., 70 µmol/L), this suggests that the observed effects may have been due to other components of the antioxidant mixture, although it does not rule out vitamin C acting synergistically with these components.

Eight of the 10 RCTs investigating oxidative burst activity and/or phagocytosis were carried out in healthy participants or athletes [19,28,29,31,35,36,37,38]. However, only one of these studies assessed baseline vitamin C status, which was already saturating [35]. It should also be noted that neutrophils saturate at lower vitamin C levels than plasma [50]. Therefore, it is unlikely that supplementing healthy volunteers or athletes with additional vitamin C over and above their normal baseline levels would have an effect on neutrophil function [51]. Exercise trials can also be complicated in that, depending on the exercise intensity, the effects on the immune system can vary such that moderate exercises may have immunopotentiating effects while intense exercise is potentially immunosuppressive [52]. Although two combination trials showed positive effects of supplementation on neutrophil function in athletes [35,39], with combination studies, it is not always possible to determine which of the component(s) is having the effects, or if there are synergistic interactions occurring between the components. 

Four of the RCTs administered intravenous vitamin C in hospital or outpatient settings, three of which saw an effect of the intervention on neutrophil chemotaxis and apoptosis [25,26,27,33]. Intravenous vitamin C is known to provide significantly higher plasma concentrations of vitamin C than oral administration [53]. Anderson et al. showed that administration of intravenous vitamin C (1000 mg once daily) was associated with improved neutrophil chemotaxis in asthmatic patients. Maderazo et al. [27] reported an increase in plasma vitamin C status and enhanced neutrophil chemotaxis in trauma patients administrated the combination of vitamins C and E. Ferron-Celma et al. [33] observed a decrease in pro-apoptotic enzymes and an increase in anti-apoptotic proteins in septic patients administered vitamin C alone. The forth study was carried out in healthy children undergoing elective surgery and although their baseline vitamin C levels were not assessed, these may have already been adequate [26]. Furthermore, the vitamin C was administered as a single dose only (10 mg/kg bodyweight), which is less likely to have an effect than repeated dosing [54]. 

There are a number of limitations of using the RCT paradigm to assess the effectiveness of nutrients such as vitamin C [55]. RCTs were specifically developed and designed to test the safety and efficacy of pharmaceutical drugs, not nutrients. For example, it is not possible to have a true placebo group in RCTs of nutrients as all of the participants will be consuming variable amounts of the nutrient of interest. Of note, a number of the assessed studies did not control for dietary intake or the participants were asked to maintain their normal diet. More than half of the RCTs included in this review did not assess baseline plasma vitamin C status, and a majority of the studies were carried out in healthy individuals, who likely already had adequate plasma vitamin C status prior to beginning the supplementation, thus negating any effect of supplementation. Only one study measured the vitamin C content of the neutrophils pre- and post-supplementation [32]. There was also variability in the analysis of the neutrophil functions, with the chemotaxis, enzyme activity, and apoptosis assays being carried out with isolated cells in buffer or culture media, and the phagocytosis and/or oxidative burst assays being carried out with either whole blood or isolated cells. Furthermore, two of the studies used only a single dose of the vitamin [26,28]. Because vitamin C is water soluble, it is cleared rapidly from circulation by the kidneys, with a half-life of approximately two hours, therefore a regular intake is required to maintain adequate levels [53]. Finally, smoking status is well known to impact on vitamin C status and requirements, due to enhanced oxidative stress [43,49], however, this was not taken into account in a majority of the studies.

## 5. Conclusions

Overall, 44% of the RCTs assessed in this review showed effects of vitamin C supplementation on various neutrophil functions. The studies were very heterogeneous, comprising different participant cohorts (athletes, hospitalized patents or healthy volunteers) and different dosing regimens (oral or intravenous, monotherapy or multi-supplements, synthetic or food-derived, and from one-off to many months in duration). There were also a number of limitations inherent in the design of many of these RCTs. Unlike drug trials, evidence indicates that RCTs of vitamin C supplementation will be more likely to have a positive effect in participants who are suboptimal or deficient in the vitamin at baseline [55]. Therefore, future RCTs should incorporate prescreening of potential participants for low vitamin C status or utilize cohorts known to have low vitamin status, such as hospitalized patients. The effects of vitamin C administration on more recently discovered functions of neutrophils, such as the formation of neutrophil extracellular traps, should also be explored in future studies based on promising in vitro and preclinical data [16]. Meta-analyses have indicated that vitamin C intakes of at least 200 mg/day can decrease the risk of acquiring respiratory infections [56,57], however, gram doses of vitamin C are required once an infection has taken hold, due to increased requirements for the vitamin [10,58]. Therefore, future RCTs should also comprise appropriate vitamin C dosing for the specific cohort under investigation.

## Figures and Tables

**Table 1:** Inclusion and exclusion criteria.

Inclusion	Exclusion
Randomized Controlled Trial Peer-reviewed publication Human Subjects Full-text access English papers Any of the following neutrophil functions as primary or secondary outcomes: Migration/chemotaxis/motility Phagocytosis Oxidative burst Enzyme activity Apoptosis/clearance Necrosis/necrotic cell death	Participants with existing neutrophilic dysfunction disorders Review articles

**Table:** (**a**). Characteristics of vitamin C-only supplementation studies.

Reference	Title of Study	Location	Trial Setting	Time Frame of Study	Neutrophil Function Assessed
Anderson et al. (1983) [25]	Ascorbic acid in bronchial asthma	Africa	Hospital Paediatric Respiratory Clinic	6 months	Chemotaxis, phagocytosis (isolated cells)
Charlton et al. (1987) [26]	Neutrophil mobility during anaesthesia in children. A trial for ascorbate premedication.	United Kingdom	Surgical Hospital	1 day	Chemotaxis (isolated cells)
Maderazo et al. (1991) [27]	A randomized trial of replacement antioxidant vitamin therapy for neutrophil locomotory dysfunction in blunt trauma	United States	Hospital	1 week	Chemotaxis (isolated cells)
Davison and Gleeson (2005) [28]	Influence of acute vitamin C and/or carbohydrate ingestion on hormonal, cytokine, and immune responses to prolonged exercise.	United Kingdom	Laboratory	3 weeks	Oxidative burst (whole blood)
Davison and Gleeson (2006) [29]	The effect of 2 weeks vitamin C supplementation on immunoendocrine responses to 2.5 h cycling exercise in man.	United Kingdom	Laboratory	2 weeks	Oxidative burst (whole blood)
Fuller et al. (2000) [30]	The effect of vitamin E and vitamin C supplementation on LDL oxidizability and neutrophil respiratory burst in young smokers	United States (North Carolina)	Community	8 weeks	Oxidative burst (isolated cells)
Krause et al. (2001) [31]	Effect of vitamin C on neutrophil function after high-intensity exercise	Austria	Outdoor Biathlon	1 week	Phagocytosis; oxidative burst (isolated cells and whole blood)
Nieman et al. (1997) [19]	Vitamin C supplementation does not alter the immune response to 2.5 h of running	United States (North Carolina)	Human Performance Laboratory	8 days	Phagocytosis; oxidative burst (whole blood)
Sureda et al. (2004) [32]	Hypoxia/reoxygenation and vitamin C intake influence NO synthesis and antioxidant defences of neutrophils.	Spain	Not specified	1 week	Enzyme activity (isolated cells)
Ferron-Celma et al. (2009) [33]	Effects of vitamin C administration on neutrophil apoptosis in patients after abdominal surgery.	Spain	Digestive Surgery Department	6 days	Apoptosis (isolated cells)

**Table:** (**b**). Characteristics of combination supplementation studies.

Reference	Title of Study	Country	Trial Setting	Time Frame of Study	Neutrophil Function Assessed
Herbaczynska-Cedro et al. (1995) [34]	Supplementation with vitamins C and E suppresses leukocyte oxygen free radical production in patients with myocardial infarction	Poland	Hospital	2 weeks	Oxidative burst (isolated cells)
Robson et al. (2003) [35]	Antioxidant supplementation enhances neutrophil oxidative burst in trained runners following prolonged exercise.	South Africa	Laboratory	7 weeks	Oxidative burst (whole blood)
Hunter et al. (2012) [36]	Consumption of gold kiwifruit reduces severity and duration of selected upper respiratory tract infection symptoms and increases plasma vitamin C concentration in healthy older adults	New Zealand	Community	20 weeks	Phagocytosis (whole blood)
Nieman et al. (2011) [37]	Ingestion of micronutrient fortified breakfast cereal has no influence on immune function in healthy children: A randomized controlled trial	United States (North Carolina)	Community	8 weeks	Phagocytosis; oxidative burst (whole blood)
Wolvers et al. (2006) [38]	Effect of a mixture of micronutrients, but not of bovine colostrum concentrate, on immune function parameters in healthy volunteers: a randomized placebo-controlled study.	The Netherlands	Unilever Food and Health Research Institute	12 weeks	Phagocytosis; oxidative burst (whole blood)
Tauler et al. (2002) [39]	Diet supplementation with vitamin E, vitamin C and B-carotene cocktail enhances basal neutrophil antioxidant enzymes in athletes.	Spain	Not specified	12 weeks	Enzyme activity (isolated cells)

**Table:** (**a**). The participant characteristics and interventions used, the frequency of intervention and the route of administration for vitamin C-only studies.

References	Participant Characteristics			Intervention and Dose Administered	Frequency of Intervention	Route of Administration
	Number (n)	Mean Age (Years)	Gender (% Women)			
Anderson et al. (1983) [25]	*n* = 16 asthmatic children	9.5	25%	Vitamin C (1000 mg/day) with standard anti-asthma chemoprophylaxis (SAC) **OR** SAC only	Once daily for six months	Intravenous
Charlton et al. (1987) [26]	*n* = 20 surgical patients	10	-	Vitamin C (10 mg/kg; mean = 363 mg) **OR** placebo	One-off	Intravenous
Maderazo et al. (1991) [27]	*n* = 46 trauma patients	24	21%	Vitamin C (200 mg/day) **OR** vitamin E (50 mg/day) **OR** both **OR** placebo	Once daily for one week	Intravenous
Davison and Gleeson (2005) [28]	*n* = 6 healthy athletes	25	0%	Vitamin C (3400 mg) **OR** carbohydrate **OR** both **OR** placebo	One-off for each intervention (crossover study)	Oral
Davison and Gleeson (2006) [29]	*n* = 9 healthy athletes	26	0%	Vitamin C (1000 mg/day) **OR** placebo	Once daily for two weeks	Oral
Fuller et al. (2000) [30]	*n* = 30 healthy smokers	20	73%	Vitamin C (1000 mg/day) **OR** vitamin E (400 IU/day) **OR** both **OR** placebo	Once daily for eight weeks	Oral
Krause et al. (2001) [31]	*n* = 10 healthy adults	29	0%	Vitamin C (2000 mg/day) **OR** none	Once daily for one week	Oral
Nieman et al. (1997) [19]	*n* = 12 healthy athletes	41	25%	Vitamin C (1000 mg/day) **OR** placebo	Once daily for eight days	Oral
Sureda et al. (2004) [32]	*n* = 7 healthy divers	-	0%	Vitamin C (1000 mg/day) **OR** placebo	Once daily for one week	Oral
Ferron-Celma et al. (2009) [33]	*n* = 20 surgical patients	67	45%	Vitamin C (450 mg/day) **OR** placebo	Once daily for six days post-operative	Intravenous

**Table:** (**b**). The participant characteristics and interventions used, the frequency of intervention and the route of administration for combination studies.

References	Participant Characteristics			Intervention and Dose Administered	Frequency of Intervention	Route of Administration
	Number (n)	Mean Age (Years)	Gender (% Women)			
Herbaczynska-Cedro et al. (1995) [34]	*n* = 45 cardiac patients	59	13%	Vitamins C and E (600 mg/day) **OR** conventional treatment only	Once daily for two weeks	Oral
Robson et al. (2003) [35]	*n* = 12 healthy athletes	30	50%	Multivitamin supplement: vitamin C content 60 mg/day **AND** antioxidant supplement: vitamin C content 900 mg/day **OR** placebo	Once daily for one week	Oral
Hunter et al. (2012) [36]	*n* = 32 healthy elderly	71	63%	2 fresh Gold kiwifruit **AND** 2 freeze dried Gold kiwifruit (comprising total of ~360 mg vitamin C) **OR** 2 freeze dried bananas	Once daily for four weeks; crossover (8 weeks washout)	Oral
Nieman et al. (2011) [37]	*n* = 65 healthy children	10	43%	Cereal fortified with micronutrients **PLUS**: Low: vitamin C content of 0.8 mg/day Medium: vitamin C content of 20 mg/day High: vitamin C content of 100 mg/day	Once daily for two months	Oral
Wolvers et al. (2006) [38]	*n* = 131 healthy volunteers	57	68%	Micronutrient mix (with vitamin C ~375 mg/day) **OR** bovine colostrum **OR** both **OR** placebo	Once daily for ten weeks	Oral
Tauler et al. (2002) [39]	*n* = 20 healthy athletes	23	0%	Antioxidant cocktail (vitamin E and β-carotene) **PLUS** vitamin C 500 mg/day (only in last 15 days) **OR** placebo	Once daily for three months (only last 15 days for vitamin C)	Oral

**Table 4:** Measurement of plasma vitamin C concentrations and control of diet and smoking status within studies.

References	Mean Plasma Vitamin C Levels		Diet Control	Smoking Status
	Baseline	Post-Intervention		
**Vitamin C-Only Studies**				
Anderson et al. (1983) [25]	~61 µmol/L	~137 µmol/L	Not controlled	-
Charlton et al. (1987) [26]	-	-	Not controlled	-
Maderazo et al. (1991) [27]	~51 µmol/L (~25 µmol/L day 1)	~54 µmol/L	Nutrition by mouth or feeding tube as needed (parenteral nutrition excluded until end of study)	-
Davison and Gleeson (2005) [28]	-	-	24-hour food diary of diet prior to exercise trials and maintained during trials	-
Davison and Gleeson (2006) [29]	-	92 µmol/L	24-hour food diary prior to exercise trials and maintained during trials	Non-smokers
Fuller et al. (2000) [30]	39 µmol/L	62 µmol/L	3-day food record completed and maintained usual diet	All smokers
Krause et al. (2001) [31]	-	-	not controlled	-
Nieman et al. (1997) [19]	-	-	7-day food record, carbohydrate intake ~60% of total energy, moderate vitamin C intake (~100 mg/day); refrained from nutrient supplement use	-
Sureda et al. (2004) [32]	30 µmol/L	80 µmol/L	7-day 24-hour recall of dietary intake and maintained during trials (Mediterranean diet)	-
Ferron-Celma et al. (2009) [33]	-	-	Not controlled	-
**Combination Studies**				
Herbaczynska-Cedro et al. (1995) [34]	38 µmol/L	77 µmol/L	Not controlled	58% smokers
Robson et al. (2003) [35]	~70 µmol/L	~90 µmol/L	Dietary intake recorded a week before exercise trials and maintained	-
Hunter et al. (2012) [36]	-	73 µmol/L	Refrained from consumption of vitamin C supplements, kiwifruit and kiwifruit products	Non-smokers
Nieman et al. (2011) [37]	-	-	3-day food record pre-study, at 1 month and at 2 months	-
Wolvers et al. (2006) [38]	~37 µmol/L	~70 µmol/L	Maintained Dutch dietary habits	Non-smokers
Tauler et al. (2002) [39]	~57 µmol/L	~94 µmol/L	Not controlled	-
